# Subjective and objective assessment of oxaliplatin-induced peripheral neuropathy

**DOI:** 10.1186/s40064-015-1646-7

**Published:** 2015-12-30

**Authors:** Yoichiro Yoshida, Ai Mogi, Teppei Yamada, Naoya Aisu, Taisuke Matsuoka, Daibo Kojima, Syu Tanimura, Tomoko Koganemaru, Mayumi Oda, Mahiru Fukuda, Fumiaki Kiyomi, Keita Noda, Keiji Hirata, Yuichi Yamashita

**Affiliations:** Department of Gastroenterological Surgery, Fukuoka University Faculty of Medicine, 7-45-1 Nanakuma, Jonan-ku, Fukuoka, 814-0180 Japan; Division of Oncology, Hematology and Infectious Diseases, Department of Internal Medicine, Fukuoka University Faculty of Medicine, Fukuoka, Japan; Nursing Department, Fukuoka University Hospital, Fukuoka, Japan; Academia, Industry and Government Collaborative Research Institute of Translational Medicine for Life Innovation, Fukuoka University, Fukuoka, Japan; Clinical research assist center, Fukuoka University Hospital, Fukuoka, Japan; Department of Surgery 1, University of Occupational and Environmental Health, Kitakyushu, Japan

**Keywords:** Adverse event, Chemotherapy, Colorectal cancer, Oxaliplatin, Neuropathy

## Abstract

Numbness and pain are currently evaluated using subjective methods such as the visual analog scale (VAS). However, because assessment of pain can vary greatly depending on the mood and physical state of the patient at the time of assessment, it is best to evaluate pain objectively. pain vision PS-2100 (PV) is an analytical instrument that was designed to quantitatively and objectively assess sense perception and nociception in patients. The present study examined the correlation of subjective and objective assessment of oxaliplatin-induced peripheral neuropathy (PN) using VAS and PV, respectively. The mean VAS and PV scores of PN were 20.5 (range 0–100) and 27.9 (range 0–416), respectively. The partial correlation coefficient was 0.274 (p = 0.0003). No strong correlation was observed between the results and a weak correlation was observed between VAS and PV.

## Background

Oxaliplatin-induced peripheral neuropathy (PN), for which there is no firmly established treatment till date, is a critical factor that makes maintenance of chemotherapy difficult (Lehky et al. 2004; Cersosimo 2005; Gamelin et al. 2008). Prevention and amelioration of oxaliplatin-induced PN are very important in improving the patient’s quality of life and encouraging continuation of therapy. However, at present, there are no effective treatments or preventive measures for oxaliplatin-associated neuropathy.

Pain intensity can be measured by visual analogue (VAS), numerical rating (NRS), or verbal rating (VRS) scales. VAS is one of the oldest, easiest and best validated measures to assess pain (Huskisson 1974). In verbal rating the patients choose one of the given verbal descriptors of the intensity of pain they feel. VRS can be used for both intensity and unpleasantness. The McGill pain questionnaire (MPQ), and the short form of it (SF-MPQ) are the most frequently used self-rating instruments for pain measurement and also often used in treatment trials (Melzack 1987). Both MPQ and SF-MPQ provide data on the various sensory and affective dimensions of pain, but they are not specifically designed to assess neuropathic pain and their translations in languages other than english need further validations. Of the scales designed for neuropathic pain assessment, the symptom score scale has only been used in diabetic neuropathy (Kvinesdal et al. 1984), and the neuropathic pain scale does not include important items such as paroxysmal pain and numbness (Galer and Jensen 1997). Both the Leeds assessment of neuropathic symptoms and signs (LANSS scale) and neuropathic pain questionnaire have been developed to differentiate neuropathic from nociceptive pain patients, rather than tools for quantitative assessment (Bennett 2001; Krause and Backonja 2003); these scales have only been preliminarily validated because they are recent and not yet widely used.

Numbness and pain are currently evaluated using subjective methods such as the VAS (McCormack et al. 1988; DeLoach et al. 1998). VAS is used in epidemiologic and clinical research to measure the intensity or frequency of various symptoms (Paul-Dauphin et al. 1999) including peripheral neuropathy by diabetes (Daousi et al. 2004; Bril et al. 2011) and chemotherapy (Liu and Wang 2012; Yang et al. 2012). However, because assessment of pain can vary greatly depending on the mood and physical state of the patient at the time of assessment, it is best to evaluate pain objectively. Determination of pain by VAS is associated with a margin of error of approximately ± 20 mm (DeLoach et al. 1998). Therefore, a method for objective assessment is also required to evaluate drugs designed to ameliorate PN.

Recently, pain vision™ PS-2100 (PV; Nipro Co., Osaka, Japan) was developed and introduced in clinical practice (Matsumura et al. 2012; Lee et al. 2014; Ohtori et al. 2014; Hiraki et al. 2014; Kim et al. 2014; Fukada et al. 2011). PV is an analytical instrument that was designed to quantitatively and objectively assess sense perception and nociception in a patient. In addition to the advantage that PV can evaluate pain in a relatively short time, it can also evaluate pain without causing additional pain to patients. Although it is used in the field of anesthesiology, there have been no reports concerning its use for the assessment of oxaliplatin-induced PN. Objective method is distinguished four different levels (Cruccu et al. 2004): (A) laboratory tests that use quantitative tools and measure an objective response; (B) quantitative sensory testing, a measure that despite using quantitative, graded stimuli inevitably relies on the patient’s evaluation; (C) bedside examination, which relies on the physician’s experience and the patient’s ability and willingness to collaborate; and (D) pain questionnaires, tools that depend entirely on the patient. Therefore, PV is classified into (B). Because individual pain thresholds are evaluated first for accurate subsequent measurement with the device, pain intensity can be quantitatively compared among patients. Therefore, this device enables a more objective evaluation when compared with other commonly used methods.

In this study, we evaluated the correlation of subjective and objective assessment of oxaliplatin-induced PN with VAS and PV, respectively.

## Methods

### Patients

This study was approved by Fukuoka University Hospital’s Institutional Review Board (No.13-4-07) and was performed between April 2014 and August 2014.

Patients with histologically proven metastatic and unresectable colorectal adenocarcinomas who had not undergone chemotherapy or who had completed adjuvant chemotherapy during the last 6 months were enrolled in the study. Patients were excluded if they had a mental disorder or poor mental health that made it impossible to understand the concepts of VAS and PV. Patients were also excluded if they had any peripheral sensory neuropathy or musculoskeletal pain before chemotherapy that may disrupt the measurement of quantitative pain. All patients provided written informed consent.

### Chemotherapy

A total of 58 patients with advanced or recurrent colorectal cancer (CRC) who received XELOX+bevacizumab therapy (7.5 mg/kg bevacizumab and 130 mg/m^2^ oxaliplatin on day 1 and 2000 mg/m^2^ capecitabine on days 1–14, every 3 weeks) or XELOX therapy (130 mg/m^2^ oxaliplatin on day 1 plus 2000 mg/m^2^ capecitabine on days 1–14, every 3 weeks) (Yoshida et al. 2015a, b) at the Department of Gastroenterological Surgery, Fukuoka University Hospital between April 2014 and August 2014 were included.

### Visual analog scale (VAS)

VAS is a simple and commonly used method for evaluating variations in pain intensity. Subjects are instructed to indicate the intensity of pain at rest and during mobilization by marking on a 100-mm horizontal line anchored with ‘‘0 (no pain)’’ on the left edge and ‘‘100 (worst imaginable pain)’’ on the right edge.

### Pain vision

The PV system was developed as a medical device to evaluate pain intensity as a numerical value. The principle of measurement of this system is to compare a unique electrical stimulation with the pain that the patient is experiencing. An electrical stimulation without pain, whose intensity is equivalent to that of the pain experienced by the patient, is applied, and the current value of this electrical stimulation is defined as the “pain-equivalent current.” The sensitivity (threshold) of the patient for the electrical stimulation is defined as the “minimum perceived current,” which is intended to eliminate variations between individuals. Using these two values, pain intensity is defined by the following formula:

Pain intensity = (pain-equivalent current−minimum perceived current)/minimum perceived current × 100.

An electrode is attached to the medial side of the upper arm. An electrical current is applied (50 Hz; 0–150 uA rms; pulse width: 0.3 ms), and the stimulation is increased. The patient is instructed to press a button when she/he perceives this stimulation for the first time; the current at this point is defined as the “minimum perceived current” value. As the stimulation current is increased, the patient is instructed to press the switch when she/he feels that the intensity of the stimulation current is equivalent to that of the pain she/he is experiencing. The current at this point is defined as the “pain-equivalent current” value. Using the values obtained, “pain intensity” is calculated using the aforementioned formula. When there is no pain, the value is 0, and it increases according to the degree of pain, with no upper limit. Each measurement is simple and can be completed within a few minutes.

### Statistical analyses

Data were collected and analyzed using SAS Version 9.3 (SAS Institute, Cary, North Carolina, USA). To investigate the reliability of the device in terms of internal consistency, we assessed the QPD score twice. Data are reported as mean ± standard deviation (SD), median (interquartile range 25–75 %), or number of participants (percentages). Partial correlation analysis was performed to estimate the relationship between PV and VAS after adjusting for sex and subject. Namely, the correlation coefficient between PV and VAS was computed using residual values of the mixed-effects model including sex as a fixed effect and subject as a random effect. P values of 0.05 or less were considered statistically significant.

## Results

Between April and August 2014, a total of 64 patients received chemotherapy for metastatic CRC. Six patients were excluded according to the selection criteria (five patients with ECOG PS 2 were excluded, and one patient was excluded because of inadequate hematological, renal, and liver functions.). A half of patients had any neuropathic pain therapy (duloxetine two patients, pregabalin eight patients, tramadol 12 patients and oxycodone seven patients). The final cohort included 40 men and 18 women ranging in age from 43 to 80 years (median age, 65 years). The patient characteristics are presented in Table 1.

**Table 1:** Baseline characteristics of patients who received chemotherapy

XELOX+BV/XELOX	40/18
Median age (range)	65 (43–80)
Male/female	68.9/31.1 %
ECOG PS 0/1	81.0/19.0 %
Primary tumor colon/rectum	53.4/46.6 %
Oxaliplatin	1751 mg/body (345–5903)

Among the included patients, 81.0 % had ECOG PS 0 at baseline, with the liver being the most common site of metastasis. The median cumulative amount of oxaliplatin was 1751 mg/body (345–5903), and the number of cycles was 10.

Grade 3 or higher hemotoxicity and grade 3 or higher non-hematological toxicity were noted in 12.5 and 17.4 % of patients, respectively.

**Fig. 1:**
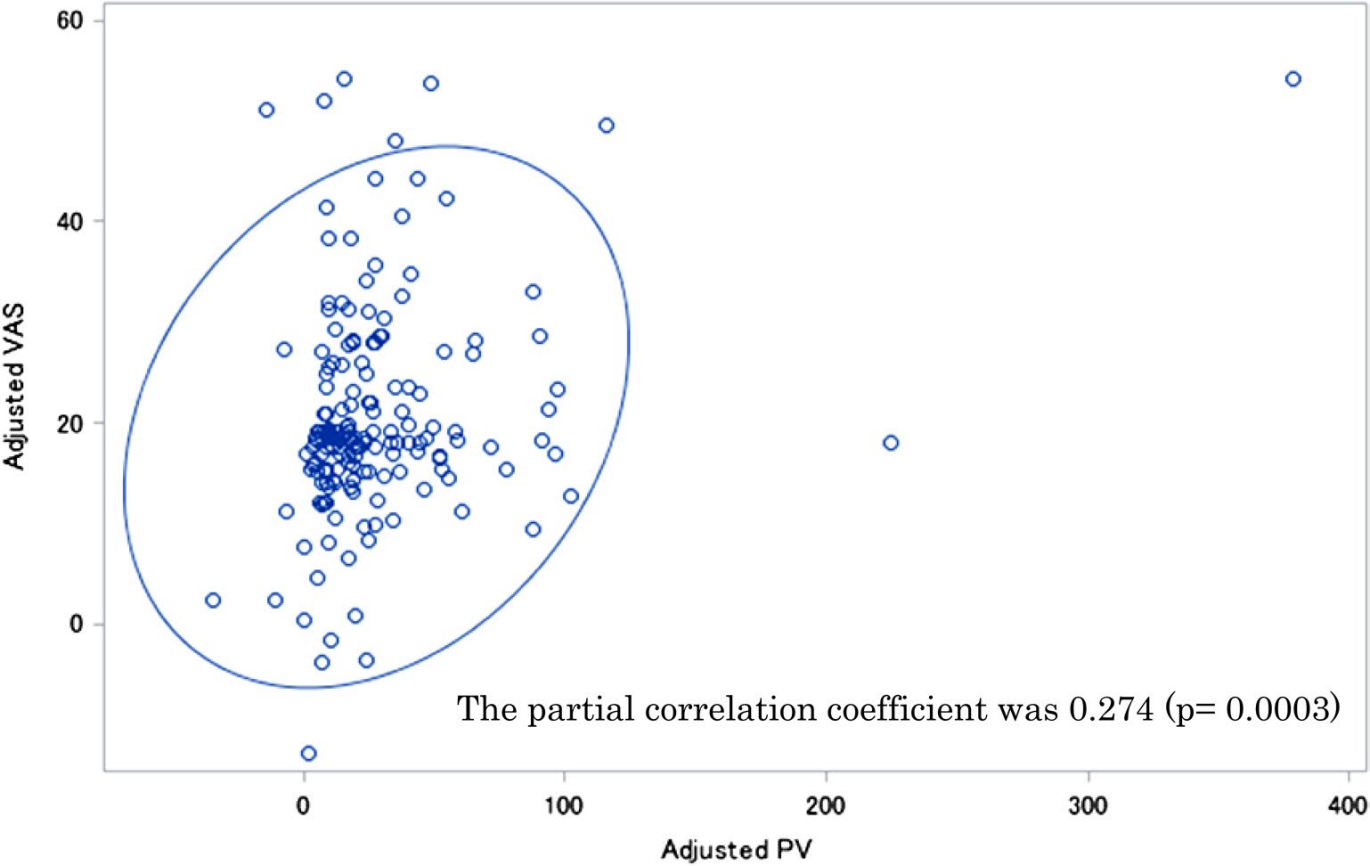
Correlation coefficient between PV and VAS adjusted for sex and subject

PN was assessed using both VAS and PV a total of 173 times. The mean VAS and PV scores of PN were 20.5 (range 0–100) and 27.9 (range 0–416), respectively. The partial correlation coefficient after adjusting for sex and subject was 0.274 (p = 0.0003) (Fig. 1).

## Discussion

VAS is one of the most common methods used for the evaluation of pain (McCormack et al. 1988). The NRS and the 4-point VRS are difficult to use when evaluating PN because they cannot distinguish small changes in pain; in contrast, VAS can distinguish changes in pain with the highest sensitivity (Seymour 1982). VAS is a subjective method of grading pain that the patient is experiencing currently in comparison with the most intense pain that the patient has ever experienced (Babul et al. 1993). Because of the ease of its use, VAS became a popular tool for quantifying pain relief and pain intensity. It has been found to be a valid and reliable means of assessing pain, depression, anxiety, and mood (McCormack et al. 1988). VAS tends to focus only on pain intensity, with an increased risk of over-simplification of the experience (Bonica et al. 1990). Furthermore, actual measurements are relative only to the individual being assessed. Identical stimuli applied to different individuals can yield markedly different scores.

A newly developed device, PV, has recently been used for the quantitative analysis of pain perception and sensation, measuring pain intensity as the “degree of pain.” In clinical practice, this method has been used for evaluating not only chronic pain, such as fibromyalgia (Osada et al. 2011) or lower back pain due to spondylolisthesis (Lee et al. 2014), but also for acute pain caused by the removal of adhesive wound dressing materials (Matsumura et al. 2012). Previous studies have shown that PV is a useful device that can evaluate pain objectively in various fields and for the quantitative assessment of sensory nerve dysfunction (Baden et al. 2011; Okamoto et al. 2013; Seno et al. 2011). This system is based on the provision of alternative painless sensory stimulation equivalent to pain (through the stimulation of sensory nerve fibers Aβ and Aδ) and the measurement of the intensity of the stimulation. However, there is no report of use of PV to evaluate PN. Prevention and improvement of oxaliplatin-induced PN is very important to improve the patient’s quality of life and to encourage continuation of treatment. However, at present, there are no effective treatments or preventive measures for oxaliplatin-associated neuropathy.

To the best of our knowledge, no previous study has investigated oxaliplatin-induced PN quantitatively using electrical stimulation. The limitation of this study include only VAS as a subjective evaluation that did not allow adequate assessment of PN. Accordingly, neuropathic pain questionnaire, leeds assessment of neuropathic symptoms and signs scale and Douleur Neuropathique en four Questions needs to be performed using well-matched groups of patients to confirm our findings. We believe that the effect of the drug for oxaliplatin-induced PN should be evaluated objectively.

## Conclusions

Although both assessments evaluated the same events, no strong correlation was observed between the results and a weak correlation was observed between VAS and PV. These results suggest that because VAS and PV each measure different factors, both are needed to evaluate oxaliplatin-induced PN with the aim of aiding treatment. These findings are expected to help in the amelioration of PN through the use of objective assessment and to support future clinical trials associated with oxaliplatin-induced PN.
